# Recent Advances in Immune-Mediated Cerebellar Ataxias: Pathogenesis, Diagnostic Approaches, Therapies, and Future Challenges—Editorial

**DOI:** 10.3390/brainsci13121626

**Published:** 2023-11-24

**Authors:** Mario Manto, Hiroshi Mitoma

**Affiliations:** 1 Service de Neurologie, Médiathèque Jean Jacquy, CHU-Charleroi, 6000 Charleroi, Belgium; 2 Service des Neurosciences, Université de Mons, 7000 Mons, Belgium; 3 Department of Medical Education, Tokyo Medical University, Tokyo 160-8402, Japan; mitoma@tokyo-med.ac.jp

**Keywords:** cerebellum, cerebellar ataxias, immunity, antibodies, paraneoplastic, immune tolerance

## Abstract

The clinical category of immune-mediated cerebellar ataxias (IMCAs) has been established after 3 decades of clinical and experimental research. The cerebellum is particularly enriched in antigens (ion channels and related proteins, synaptic adhesion/organizing proteins, transmitter receptors, glial cells) and is vulnerable to immune attacks. IMCAs include various disorders, including gluten ataxia (GA), post-infectious cerebellitis (PIC), Miller Fisher syndrome (MFS), paraneoplastic cerebellar degeneration (PCD), opsoclonus myoclonus syndrome (OMS), and anti-GAD ataxia. Other disorders such as multiple sclerosis (MS), acute disseminated encephalomyelitis (ADEM), Behçet disease, and collagen vascular disorders may also present with cerebellar symptoms when lesions are localized to cerebellar pathways. The triggers of autoimmunity are established in GA (gluten sensitivity), PIC and MFS (infections), PCD (malignancy), and OMS (infections or malignant tumors). Patients whose clinical profiles do not match those of classic types of IMCAs are now included in the spectrum of primary autoimmune cerebellar ataxia (PACA). Recent remarkable progress has clarified various characteristics of these etiologies and therapeutic strategies in terms of immunotherapies. However, it still remains to be elucidated as to how immune tolerance is broken, leading to autoimmune insults of the cerebellum, and the consecutive sequence of events occurring during cerebellar damage caused by antibody- or cell-mediated mechanisms. Antibodies may specifically target the cerebellar circuitry and impair synaptic mechanisms (synaptopathies). The present Special Issue aims to illuminate what is solved and what is unsolved in clinical practice and the pathophysiology of IMCAs. Immune ataxias now represent a genuine category of immune insults to the central nervous system (CNS).

The cerebellar circuitry is characterized by a high diversity of cells and antigens, being enriched in numerous extra-cellular and intra-cellular antigens (ion channels and related proteins, synaptic adhesion/organizing proteins, transmitter receptors, glial cells) [1,2]. The cerebellum is particularly vulnerable to immune attacks. Well-characterized immune-mediated cerebellar ataxias (IMCAs) include gluten ataxia (GA), post-infectious cerebellitis (PIC), Miller Fisher syndrome (MFS), paraneoplastic cerebellar degeneration (PCD), opsoclonus myoclonus syndrome (OMS), and anti-GAD ataxia [3,4,5,6]. This Special Issue discusses advances in our understanding of the pathogenesis of IMCAs and highlights lines of management, leading to a better delineation of this novel group of immune disorders.

Glial fibrillary acidic protein (GFAP) astrocytopathy (GFAP-A) represents an autoimmune corticosteroid-responsive meningoencephalitis that occurs with or without concomitant myelitis [7,8,9]. IgG against GFAP (GFAP-IgG) in the cerebrospinal fluid (CSF) is a biomarker of GFAP-A. About 50% of patients show a brain linear perivascular radial gadolinium enhancement (LPRGE) pattern which is highly suggestive of GFAP-A. Usually, patients present clinically with fever and headache in the context of meningeal syndrome. The features of movement disorders are clarified by the study of Kimura et al. [10]. The authors reviewed in detail clinical data from 87 consecutive patients with GFAP-A. Seventy-four patients (85%) showed movement disorders, including ataxia (49%), tremors (45%), myoclonus (37%), dyskinesia (2%), opsoclonus (2%), rigidity (2%), myokymia (1%), and choreoathetosis (1%). Patients exhibiting movement disorders were significantly older than those without. This study highlights that movement disorders are common in GFAP-A patients. Facing ataxia, tremor or myoclonus, the diagnosis of GFAP-A should be considered, especially in the elderly. From the immune standpoint, GFAP-specific CD8+ T cells are likely key mediators of this disorder. Although GFAP-A is often responsive to steroids, relapses may occur [11]. Administration of steroids should not be delayed.

IMCAs include not only the well-established entities reported above, but also primary autoimmune cerebellar ataxia (PACA), which corresponds to ataxic conditions suspected to be autoimmune even in the absence of specific well-characterized pathogenic antibody markers [12]. The diagnostic criteria for PACA are based on clinical symptomatology (mode of onset, pattern of cerebellar involvement, presence of other autoimmune diseases), imaging studies (MRI and, if available, MR spectroscopy showing preferential, but not exclusive, involvement of vermis) and laboratory tests (CSF pleocytosis and/or CSF-restricted IgG oligoclonal bands). Hadjivassiliou et al. report on the frontiers of PACA [13]. The early identification of rare immune ataxias is an important step since they are potentially treatable [14]. Delays in therapies may cause disabling sequelae.

Parvez and Ohtsuki discuss the mechanisms of neuroinflammation and the emergence of acute cerebellar ataxia [15]. The authors clarify the consequences of acute infections (viruses, bacteria, and fungi) upon immunity and glial reactions. They discuss how inflammation and immunity interact with essential neurophysiological mechanisms including excitability, inhibition and excitation of the main elements of the cerebellar circuit. The authors underline that inflammation triggered by infections impacts upon neurotransmission at multiple levels within the circuitry, disrupting the basic functions of the mossy fibers, the climbing fibers, the cerebellar cortex and cerebellar nuclei. Preventing or blocking neuroinflammation appears as an appealing question for the neuroscience of the cerebellum.

The brain is protected from the periphery by the blood–brain barrier (BBB), blood–CSF barrier, and blood–leptomeningeal barrier (BLMB) [16,17,18]. Entry of immune cells into the brain for immune surveillance is restricted to the blood–CSF barrier. Autoimmune responses targeting the cerebellum are accompanied by the invasion of peripheral immune cells into the brain. A breakdown in the barrier permeability is associated with the entry of peripheral immune cells into the cerebellar compartment. One of the mechanisms is the molecular mimicry between the trigger and a host protein. Hampe and Mitoma discuss the interactions between the CNS and the immune system and the constant immune surveillance exerted by both microglia and peripheral immune cells [19]. Microglia are present in the brain and spinal cord, whereas peripheral immune cells must transmigrate into the CNS either at the BBB, the blood–CSF barrier, or the BLMB. The identification of the cellular mechanisms leading to CNS invasion will likely lead to novel therapies for IMCAs [20].

The neuropathology of IMCAs has been poorly studied so far. Clark reports on the differential diagnosis between IMCAs and hereditary/sporadic ataxias from the neuropathological point of view [21,22]. Indeed, IMCAs can clinically mimic other ataxias. Inflammatory infiltrates strongly indicate an autoimmune mechanism. Subacute clinical onset, auto-antibodies, the demonstration of a neoplasm or the presence of another auto-immune disorder point towards an IMCA when inflammatory changes are minimal or absent and the pathology is limited to the cerebellum and its anatomical connections. Pathological abnormalities may not always be due to a specific auto-antibody, as cell-mediated immunity versus humoral immunity are both implicated to various degrees.

PCD, an exemplative entity within IMCAs, is discussed by Loehrer et al. (2021) [23]. PCD results from a remote effect of cancer in the absence of direct invasion by the tumor or metastases [24]. Early diagnosis is essential because it may lead to the discovery of an occult cancer [25]. Autoantibodies are varied and target either intra-cellular antigens (in nuclei or cytoplasm) or plasma membranes [26]. In most cases of PCD, the cancer is gynecological, located in the lungs or is a Hodgkin lymphoma [27]. Neurological deficits may precede the cancer by several months or a few years. Antibodies targeting the intra-cellular antigens include the anti-Yo (PCA1) antibody binding to the cerebellar protein cdr2, which inhibits c-Myc, the anti-Hu (ANNA1) antibody impacting on post-transcriptional regulation of RNA, and the anti-Ri (ANNA2) antibody binding to the NOVA family of RNA binding proteins [28,29]. Antibodies binding to plasma-membrane antigens include the PCA-Tr antibody acting on the Delta/notch-like epidermal growth factor-related receptor, mGLUR1-IgG and VGCC-IgG [30,31]. There is a consensus that the detection of antibodies should be followed by the continuous observation of the patient. The autoimmune response is presumed to be provoked when proteins restricted to immune-privileged neurons are accessible by the malignancy, triggering a cytotoxic T-cell response and/or a direct pathogenic effect of antibodies, especially when surface receptors are the antigens. One of main hallmarks in several forms of PCD is the destruction of Purkinje neurons. The clinical picture is typically a subacute cerebellar syndrome which can mimic a posterior circulation stroke or a vestibular neuronitis [32,33,34]. The cerebellar syndrome is occasionally subtle at the onset but will deteriorate over days or weeks [35]. Therapies include the eradication of the underlying cancer without delay, steroids, immunoglobulins, plasmapheresis, and maintenance immunotherapy, with the goal of stabilization in most cases. Some PCD are more responsive. Important advances in the elucidation of the pathogenesis of PCD include not only the description of antibody effects, especially those targeting cell-surface antigens, and attempts to isolate antigen-specific T-cells, but also the assessment of genetic predisposition [36,37]. Genetic alterations of Yo antigens are involved in the triggering of the immune tolerance breakdown.

Long-term depression at parallel fibers–Purkinje cells (PF-PC LTD) is a major neurobiological mechanism of cerebellar motor learning [38,39]. The dysregulation of PF-PC LTD in IMCAs is discussed by Mitoma et al. [40]. Autoantibodies against voltage-gated Ca channel (VGCC), mGluR1 and glutamate receptor delta (GluR delta) impair PF-PC LTD. The concept of LTDpathy encompasses a clinical spectrum including etiologies associated with a functional disturbance of PF-PC LTD, leading to the impairment of related adaptative behaviors, including VOR, the blink reflex, and prism adaptation. A functional impairment may anticipate neuronal loss. The early functional impairment of synaptic plasticity eventually impacts on learning and adaptation, causing a clinical deficit where dysmetria is a predominant feature [41,42].

Anti-GAD Ab have been associated with multiple neurological syndromes, including stiff person syndrome (SPS), cerebellar ataxia, and limbic encephalitis, all of which are considered to be related to a reduced GABAergic transmission [43,44]. Anti-GAD Ab have been associated with nystagmus (up/down-beat, periodic alternating nystagmus), deficits of abduction, saccadic impairments, opsoclonus and flutter [45,46]. Ophthalmoparesis has been reported in SPS [47]. Belem et al. report a 26-year-old male patient with anti-GAD ataxia presenting initially with ophthalmoplegia and showing full recovery after immunotherapy [48]. He subsequently developed progressive ataxia. Administration of methylprednisolone and immunoglobulin resolved symptoms and was followed by a drop in anti-GAD Abs titers. This is the first case of isolated ophthalmoparesis due to tonic eye deviation associated with anti-GAD antibodies without SPS. With growing evidence for ocular abnormalitites in SPS, anti-GAD-associated neurological syndromes should be included in the differential diagnosis of ophthalmoplegia [49].

This Special Issue highlights the importance of the early identification of IMCAs is daily practice. IMCAs now represent an established entity from the clinical, pathological and pathophysiological standpoint, with potentially curable disorders if therapies are initiated when the cerebellar reserve is sufficient [50]. Indeed, the cerebellar circuitry has the capacity to compensate for and restore lost functions given the abundant synaptic plasticity and the convergence of multimodal central and peripheral signals [51,52,53,54]. Potentiation of the cerebellar reserve may lead to compensation and restoration of function in cerebellar disorders. Detailed studies on the cerebellar reserve in IMCAS are warranted as they may be of neurobiological importance to a large range of neurological/neuropsychiatric conditions [55,56]. This is also applicable to latent autoimmune cerebellar ataxia (LACA), which is characterized by a slowly progressive course, the lack of an obvious autoimmune background, and difficulties in reaching a diagnosis in the absence of biomarkers for IMCAs [57,58]. IMCAs have now entered into daily neurological practice [59,60]. IMCAs represent a window to elucidate both fundamental and clinical aspects of cerebellar disorders encountered in daily practice.
